# Propagule pressure and hunting pressure jointly determine genetic evolution in insular populations of a global frog invader

**DOI:** 10.1038/s41598-018-37007-6

**Published:** 2019-01-24

**Authors:** Supen Wang, Conghui Liu, Jun Wu, Chunxia Xu, Jiaqi Zhang, Changming Bai, Xu Gao, Xuan Liu, Xianping Li, Wei Zhu, Yiming Li

**Affiliations:** 1 CAS Key Laboratory of Animal Ecology and Conservation Biology, Institute of Zoology, 1 Beichen West Road, Chaoyang District, Beijing, 100101 China; 2 University of Chinese Academy of Sciences, 19 Yuquan Road, Shijingshan, Beijing, 100049 China; 3 Nanjing Institute of Environmental Sciences under Ministry of Environmental Protection of China, No. 8 Jiang Wang Miao Street, Nanjing, 210042 PR China; 4 Key Laboratory of Sustainable Development of Marine Fisheries, Ministry of Agriculture, Yellow Sea Fisheries Research Institute, Chinese Academy of Fishery Sciences, Qingdao, 266071 China; 5 College of Resources and Environmental Science, Nanjing Agricultural University, Nanjing, 210095 China

## Abstract

Islands are often considered to be more susceptible to biological invasions and to suffer greater impacts from invaders than mainland areas, and this difference is generally attributed to differences in species introductions, ecological factors or human activities between islands and mainland areas. Genetic variation, as a good estimate of evolutionary potential, can influence the invasion process and impacts of alien species. However, few studies have compared the genetic diversity of alien species between islands and a corresponding mainland. Here, we examined the genetic variation and differentiation in feral populations (30 sampled individuals/population) of a globally invasive species (the American bullfrog, *Lithobates catesbeianus*) that was extensively farmed on 14 islands in the Zhoushan Archipelago of China and in three nearby regions on the mainland. We quantified the relative importance of propagule pressure and hunting pressures on the genetic variation of bullfrog populations and found that insular populations have greater genetic variation than their mainland counterparts. Although genetic differentiation between the populations was observed, no evidence of recent bottlenecks or population expansion in any of the tested population was found. Our results suggest that the propagule pressures of bullfrogs escaping from farms, multiple releases and hunting pressure influence the genetic variation among bullfrog populations. These results might have important implications for understanding the establishment and evolution of alien species on islands and for the management of invasive species.

## Introduction

Islands are often considered to be more susceptible to biological invasions and to suffer greater negative impacts from invaders than the mainland^1,2^. This difference is generally attributed to the facts that islands often harbor more alien species, that islands have lower biological resistance (e.g., lower competition pressures or predation pressures) to invaders than the mainland areas; or that islands experience more human disturbances than the mainland areas^3–9^, and/or that islands exhibit a lower hunting pressure^10^. Genetic variation, a good estimate of population fitness and adaptive potential, may influence the invasion process and impacts of alien species^11,12^. Greater genetic diversity is hypothesized to promote rapid evolutionary responses to selection pressures in novel environments and may therefore facilitate invasion success and range expansion^13,14^. However, few studies have compared the genetic diversity and structures of alien species between islands and the mainland^15,16^.

Identifying the factors that influence the genetic variation of alien species on islands is not only important for understanding their successful establishment and evolution but is also crucial for the prevention and eradication of such species. The introduction history (e.g., propagule pressure and residence time), island environment and human activities (e.g., hunting pressure) are likely to influence the genetic variation of insular introduced populations. Low propagule pressure (e.g., the frequency of introductions of a species to a site and the number of individuals in each introduction event) from a single introduction often leads to reductions in initial genetic diversity^12,15–17^. A small founder population only represents a portion of the genetic variation in the native populations and is more likely to result in a genetic bottleneck. In contrast, multiple introductions of a species from different source populations in the native range may facilitate the admixture of previously isolated native populations and increase the genetic variation in the invading populations^18,19^. Residence time (e.g., the time since a population became established) may reflect the time dimension of propagule pressure^20^. With increasing residence time after the first population becomes established, more propagule pressure from multiple releases (e.g., those that follow the first introduction) may be introduced into a region, which would promote genetic variation and differentiation in the invading populations in the region^20^. More or less propagule pressure from introductions to islands compared with a mainland might directly result in higher or lower initial genetic diversity in insular populations. In addition, island environments, which are characterized by a lower resistance of native species or pathogens and/or decreased hunting pressure to invaders compared with mainland areas, are more likely to reduce the mortality of insular propagules^2,6,8,10,21^ or to reduce the length of the bottleneck period following an introduction, which could lead to more genetic variation in the insular populations than in their mainland counterparts.

The American bullfrog (*Lithobates catesbeianus* = *Rana catesbeiana*, hereafter referred to as the bullfrog) is one of the world’s worst invaders^22^. Native to eastern North America, the bullfrog has been introduced to many countries and regions, including both mainland regions, such as Europe, Asia, South America and some areas of North America outside its native range, and some islands, including Vancouver Island, the Caribbean Archipelago, Crete, the Hawaiian Archipelago, the Zhoushan Archipelago, Japan, Singapore, Sri Lanka, and Indonesia^10,23,24^. Bullfrog invasions have led to population declines among some native amphibians in the invaded range through competition (bullfrogs have a high reproductive ability and high densities) with or predation (bullfrogs are generalist predators, eating anything that fits into their mouths) on native amphibians^25–28^. Bullfrogs can also spread chytrid fungus, *Batrachochytrium dendrobatidis*, which is the proximate cause of rapid amphibian declines across diverse biomes^29^. Bullfrogs are usually found in permanent still water bodies, such as ponds, pools, lakes or reservoirs^28,30^. The frog is intentionally introduced for aquaculture (frog leg trade) or as pets^31,32^. Feral bullfrog populations are established by bullfrogs escaping from farms or by frogs released by people^10,31–33^.

The well-documented bullfrog invasions in the Zhoushan Archipelago in the East China Sea and near the Chinese mainland^10,34–36^ provide an ideal model system for examining genetic variation in populations of alien species between islands and the mainland. Bullfrogs were introduced to Hansu in Hunan Province, China, for aquaculture from Cuba via a single introduction in the late 1950s^34^. Other captive bullfrog populations in China were introduced (human-aided dispersal) from Hansu during the 1980s. Because most feral populations of bullfrogs were founded by escaping individuals from temporary (0.5–2 years), low-quality enclosures (the farms usually stopped raising frogs because there was no economic benefit due to the large number of escaping bullfrogs)^10,36^, information on the number of bullfrogs raised and the number of farms in a region has been summarized in great detail^10,35^. The number of bullfrogs raised in a region may be a surrogate for the propagule pressure of local introductions because the local invasion success increases with the number of bullfrogs raised on farms^10,36^. Furthermore, due to recent introductions, the residence time for each invaded site can be determined^35^. This availability of data provides a good opportunity to evaluate the effect of introduction history on genetic diversity and differentiation in alien species.

We hypothesize that propagule and hunting pressures have a profound impact on the genetic variation of bullfrog populations, leading to two major predictions concerning the expected distribution of the nuclear microsatellite diversity of bullfrog populations in the island system of the Zhoushan Archipelago. First, island bullfrog populations will exhibit enhanced genetic diversity relative to mainland populations. Second, the genetic diversity of island bullfrog populations will be influenced the number of bullfrogs raised, the residence time, the number of bullfrog farms and the hunting pressure.

## Materials and Methods

### Ethics Statement

The methods were designed based on the Good Experimental Practices adopted by the Institute of Zoology, Chinese Academy of Sciences, China. All experimental procedures and animal collection were approved by the Animal Ethics Committee at the Institute of Zoology, Chinese Academy of Sciences, China (Permit Number: IOZ10013).

### Study area

**Figure 1:**
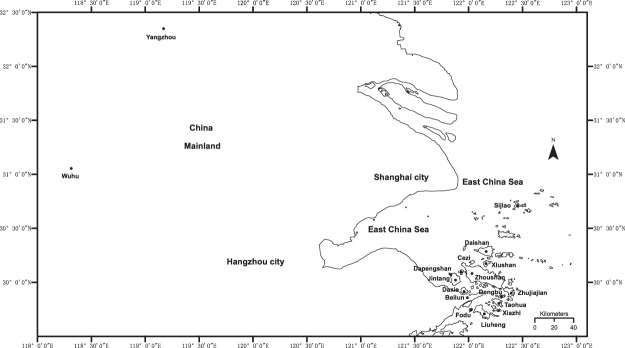
Sampled areas for bullfrog invasions in the Zhoushan Archipelago and regions in mainland China. The closed circles indicate the sampling site.

We included 14 islands of the Zhoushan Archipelago: Daxie, Fodu, Liuheng, Xiazhi, Taohua, Dengbu, Zhujiajian, Zhoushan, Cezi, Jintang, Depengshan, Xiushan, Daishan and Sijiao (Fig. 1).We also conducted this study in three bullfrog-invaded regions on the nearby mainland: Beilun, Wuhu, and Yangzhou (Fig. 1). The Zhoushan Archipelago is located in the East China Sea and is part of the Zhejiang Province. Data on the area of the islands were obtained from Wang^37^.

### Number of bullfrogs raised in an enclosure, number of farms, residence time and island area

We obtained information on the owners of the bullfrog farms from the Fisheries Bureau for the counties where the sampling sites were located. According to a regulation in China, a permit issued from the bureau is needed before a bullfrog farm can be operated. We then visited each owner to collect information on the number of bullfrogs raised on the farm and the periods during which bullfrogs were raised using a questionnaire survey^10^. The location of each farm was determined by GPS (geographic positioning system; Magellan eXplorist 210, Santa Clara, CA, USA). Bullfrog farms are generally located near a permanent still-water body or rice fields near permanent still-water bodies (less than 1 km from a water body) because they require a water supply. We counted the number of bullfrog farms within a plot (with a radius of 1 km centered at a sampling site) for each of the sampled sites in a region or island and summed the number of bullfrogs raised on the farms. For the sampled sites where a bullfrog farm was located, the residence time was considered as the time since the farm began to raise bullfrogs. We obtained information on residence time in the sampled sites without a bullfrog farm by using questionnaire surveys (Table S1, Supporting Information)^35^. We usually interviewed two or three resident individuals living around each of these sampled water bodies. The residence time was based on the time since these individuals first saw juvenile or adult bullfrogs or heard bullfrog calls. When several interviewers gave different answers for the residence time for a bullfrog-invaded site, the average value (year) of their answers was used. The residence time for all the sampled sites in a region or island is defined as the longest reported residence time.

### Hunting pressure

We obtained information on the hunting pressure in invasive regions through the use of questionnaire surveys and field monitoring in 2011–2018^10^. For the questionnaire surveys, we interviewed three resident individuals living in the invasive region and asked “is there frog hunting activity at night.” The answers were usually “frequent hunting”, “occasional hunting” or “no hunting”, and the answers provided by the three residents in each invasive region were always consistent. For field monitoring, we recorded any human hunting activity in the invasive regions during three consecutive nights. Hunters usually used electric torches to find bullfrogs and caught them by hand or with a fishing net during the night. Therefore, we could find hunters by torchlight during the night. “Frequent hunting” was associated with higher hunting pressure. We classified hunting pressure as a dichotomous variable with the values “frequent hunting” and “no hunting”. An invasive region was considered to “frequent hunting” if the questionnaire surveys yielded the answer “frequent hunting” or hunting activity was detected in two of the three nights monitored, and all other cases were defined as no (or occasional) hunting.

### Sampling, DNA extraction and multilocus microsatellite genotyping

We randomly captured 30 postmetamorphic bullfrogs in invaded water bodies manually or with long-handled nets between May and October in 2011–2018. The distal third of the third toe on the right hind-foot from each frog was clipped. We then released the frog at its capture site. Each tissue sample from the toe clips was preserved separately in 95% ethanol in a 2-ml screw-cap microcentrifuge tube and stored at −20 °C in the laboratory.

DNA was extracted following a published procedure^38,39^. In brief, the tips of bullfrogs’ second hind toes (approximately 3 mg) were clipped and placed in a 2-ml centrifuge tube with 100 μl of lysis buffer containing 0.01 M NaCl, 0.1 M EDTA, 1 mg/ml proteinase K, 0.01 M Tris–HCl (pH 8.0) and 0.5% Nonidet P-40. The tube was vortexed for 1 min at ambient temperature, centrifuged to recover all material from the bottom of the tube, and incubated at 50 °C for 120 min and at 95 °C for 20 min. The tube was then centrifuged at 12,000 rpm for 3 min in a cold temperatures, and the extract was diluted to one tenth of its original concentration for polymerase chain reaction (PCR) amplification. The individuals were genotyped using nine nuclear microsatellite loci (BF1, BFD11 and GenBank accessions AY323928-AY323934). Sequence information for the primers and the amplification conditions used for these loci were based on previously published data^40,41^ (Table S2, Supporting Information). All the primers were tagged with 5′-fluorescein bases (TAMRA, FAM or HEX). PCR reactions were performed as previously described^41,42^. The amplification conditions consisted of an initial denaturation at 94 °C for 3 min followed by 35 cycles of 10 s at 94 °C, 30 s at the annealing temperature (Table S2), and 30 s at 72 °C and a final 10-min extension at 72 °C. The PCR products were then separated by 2% agarose gel electrophoresis. Following amplification, the PCR products were resolved using an ABI PRISM 377 DNA Sequencer (Applied Biosystems) to resolve these PCR products. GENESCAN version 3.7 (Applied Biosystems) and GeneMarker version 1.71 (SoftGenetics) were used to score the microsatellite fragments.

### Statistical analysis

We applied MICRO-CHECKER 2.2.3^42^ to quantify the scoring errors resulting from factors such as large allele dropout or stuttering and null alleles. GENEPOP version 4.0^43^ was employed to test the linkage disequilibrium and Hardy-Weinberg equilibrium. We used Bonferroni corrections to test multiple comparisons^44^.

Because the sampled bullfrogs within entire invasive regions or islands were collected, there might be some genetic structure among the sampled bullfrogs. We therefore examined the presence of genetic structure for the sampled bullfrogs within each sampled region or island using STRUCTURE version 2.3.3^45^ based on the Bayesian clustering approach. This analysis revealed no genetic structure for the sampled bullfrogs for all sampled regions and islands (≥2 sampling sites) (Fig. S1, Supporting Information). Consequently, we treated the sampled bullfrogs within each region or island as a population.

We applied GenAlEx 6.5^46^ to quantify the expected heterozygosity (*He*) and the observed heterozygosity (*Ho*), and the mean number of alleles (*Na*) for each region or island. Asymmetric migration rates were estimated using the MIGRATE-N 3.2.7 program^47^.

We examined the difference in the mean genetic diversity and allelic richness for bullfrogs between introduced regions on the mainland and the islands using a one-way ANOVA. We tested the differences in residence time, the number of bullfrogs raised and the number of farms between introduced regions on the mainland and the islands using a Wilcoxon rank sum test.

We determined the genetic divergence between regions or islands using *Dest* in the R package DEMEtics V0.8.0^48^, which is an unbiased estimator of genetic divergence for multiple loci^49^. The significance of the *Dest* results was assessed by bootstrapping with 1,000 permutations. In addition, we examined the relationship between the pairwise *Dest* and relative residence time using a Mantel test implemented in ARLEQUIN3.5 with 100,000 permutations^50^.

We tested for population bottlenecks using the program BOTTLENECK^51,52^. This software measures heterozygosity excess with respect to the mutation-drift equilibrium characteristic of population-size reductions for each population to infer recent population bottlenecks. We applied the one-tailed Wilcoxon signed rank test to determine the significance of heterozygosity excess. Two-phase mutation models were used: 90% single-step mutations and 10% multiple-step mutations with 10,000 replicates. To assess population expansion, we performed an intralocus k-test and interlocus g-test^53,54^ using KGTESTS Excel Macro^53^. The intralocus k-test estimates the *P* value of k using the one-tailed binomial distribution. The significance of the g value for expanding populations was set at *α* = 0.05^54^.

Island area and the number of bullfrogs raised were log-transformed to improve linearity. Because several predictors were collinear (Fig. S2, Supporting Information), we used multimodel inference based on information theory^55^ to evaluate the relative importance of the introduction history and hunting pressures on the expected heterozygosity. The full model was a multiple linear regression model with *He* as the response variable and with hunting pressure (frequent hunting = 1, no or occasional hunting = 0), residence time, number of bullfrogs raised and number of farms as predictors for island populations. All the models that included all possible combinations of the four (2^4^−1 = 15 models) variables were ranked. We compared the alternative models using the second-order Akaike information criterion (*AICc*)^55^ and calculated the parameter estimates and their variances based on Akaike weights. Those models that were within two AICc units of the highest-ranked models (i.e., ΔAIC ≤2)^55^ were reported. We conducted these analyses using the *lme4* and *MuMIn* packages in R version 2.15.2^56^.

## Results

### Genetic diversity in insular and mainland populations

The number of sampled sites on an island or in a region ranged from one to four bodies of water and there was no significant difference between the island and mainland sampling points (Mann-Whitney U test: *Z* = 0.066, *P* = 0.948). The number of bullfrogs sampled in each site ranged from seven to 30 individuals in an island or from five to 30 individuals in a region (Table S3, Supporting Information).

MICRO-CHECKER did not detect the presence of null alleles or scoring errors. After a Bonferroni correction, we found no patterns of linkage disequilibrium among the loci and populations (*P* > 0.05). We also found that none of the populations or loci showed significant departure from Hardy-Weinberg equilibrium after Bonferroni corrections (*P* > 0.05). The loci were polymorphic within all populations.

The expected heterozygosity (*He*) and observed heterozygosity (*Ho)* for mainland populations ranged from 0.52 to 0.56 and 0.56 to 0.67 (Table 1), respectively, and for insular populations from 0.49 to 0.73 and 0.5 to 0.76, respectively. *He* differed from *Ho* across sampled regions or islands (Paired sample t-test, *P* < 0.001). The mean number of alleles (*Na*) and effective population size (*θ*) for mainland populations ranged from 3.78 to 4.33 and 0.0022 to 0.0145, respectively, and for island populations from 4.22 to 6.78 and 0.0022 to 0.0971, respectively. Overall, the genetic diversity (*He*) and allelic richness (*Na*) for island populations were higher than those for mainland populations (one-way ANOVA, *F* = 6.482, *d.f*. = 1, *P* = 0.022 for *He*; *F* = 6.604, *P* = 0.027 for *Na*).

**Table 1:** Genetic diversity and effective population size of *L*.

Location	*He*	*Ho*	*Na*	*θ* (95CI)
**Mainland**				
Wuhu	0.56 ± 0.10	0.56 ± 0.03	4.22 ± 1.72	0.0072 (0.0011–0.0076)
Beilun	0.52 ± 0.09	0.67 ± 0.03	3.78 ± 1.56	0.0145 (0.0033–0.0204)
Yangzhou	0.54 ± 0.01	0.56 ± 0.03	4.33 ± 1.66	0.0022 (0.0005–0.0038)
**Islands**				
Dapengshan	0.62 ± 0.04	0.67 ± 0.03	4.67 ± 1.80	0.0131 (0.0037–0.0190)
Liuheng	0.72 ± 0.03	0.76 ± 0.03	6.78 ± 2.17	0.0405 (0.0483–0.0528)
Taohua	0.63 ± 0.05	0.69 ± 0.03	5.00 ± 2.40	0.0146 (0.0042–0.0159)
Dengbu	0.67 ± 0.03	0.73 ± 0.03	5.44 ± 2.35	0.0048 (0.0019–0.0061)
Zhujiajian	0.69 ± 0.03	0.74 ± 0.03	6.00 ± 1.73	0.0742 (0.0421–0.0942)
Zhoushan	0.73 ± 0.04	0.76 ± 0.03	6.56 ± 2.19	0.0971 (0.0376–0.1000)
Xiushan	0.72 ± 0.03	0.75 ± 0.03	6.78 ± 2.54	0.0905 (0.0788–0.1000)
Daishan	0.68 ± 0.04	0.72 ± 0.03	6.11 ± 3.30	0.0824 (0.0639–0.1000)
Xiazhi	0.61 ± 0.04	0.69 ± 0.03	5.33 ± 3.81	0.0121 (0.0087–0.0181)
Fodu	0.62 ± 0.06	0.67 ± 0.03	5.11 ± 2.26	0.0150 (0.0102–0.0197)
Cezi	0.64 ± 0.04	0.67 ± 0.03	5.11 ± 1.83	0.0078 (0.0029–0.0097)
Jintang	0.59 ± 0.06	0.62 ± 0.03	4.56 ± 1.59	0.0022 (0.0002–0.0042)
Daxie	0.56 ± 0.05	0.62 ± 0.03	4.22 ± 0.97	0.0030 (0.0012–0.0048)
Sijiao	0.49 ± 0.09	0.50 ± 0.03	4.22 ± 2.17	0.0030 (0.0010–0.0050)

*catesbeianaus* on the Zhoushan Archipelago and mainland, China. *He* = expected heterozygosity. *Ho* = observed heterozygosity. *Na* = mean number of alleles. *θ* = effective population size.

### Genetic differentiation, gene flow and genetic bottlenecks among insular and mainland populations

The pairwise population *Dest* differences ranged from 0.037 to 0.727 (significant α = 0.05 after Benjamini-Hochberg correction, P < 0.001 for all comparisons, Table S4, Supporting Information). These results indicated genetic differentiation between the population pairs.

Low-immigration-rate 95% CIs calculated by MIGRATE-N demonstrated that differences among populations did not differ significantly from zero, indicating that gene flow between these populations is likely lacking (Table S5, Supporting Information). We did not detect evidence for recent bottlenecks (one-tailed Wilcoxon signed rank test, *P* > 0.01) or population expansion in any of the populations (*P* > 0.05 Table S6, Supporting Information).

### Correlation between factors and genetic diversity in insular populations

The number of bullfrogs raised on farms and the number of farms around sampled water bodies ranged from 1,300 to 533,330 bullfrogs and one to four farms on each island and from 8,300 to 12,200 bullfrogs and only one farm in each region of the mainland (Table S7, Supporting Information). The residence time for the earliest invaded water body on an island was between 19 and 23 years, and that for a region of the mainland was between 15 and 27 years (Table S7, Supporting Information).

The number of bullfrogs raised on islands was higher than that in invaded regions of the mainland (Mann-Whitney U test, *z* = −2.269, *P* = 0.023), but there was no difference in the residence time between islands and regions of the mainland (*z* = −1.821, *P* = 0.069 for the number of farms; *z* = −1.204, *P* = 0.229 for the residence time).

The three most highly supported models (i.e., ΔAICc ≤2) for insular populations contained three variables: the number of bullfrogs raised, the residence time and hunting pressure (Table 2). These predictors explained a proportion of the variation in the models (*R*^2^) ranging from 0.8015 to 0.8691 for insular populations (Table 2). However, there was moderate model selection uncertainty across models (*Wi* = 0.206 to 0.304) (Table 2).

**Table 2:** The best models (i.e. ΔAIC ≤ 2) containing factors influencing the genetic variation (*He*) of *L. catesbeianaus* in the Zhoushan Archipelago.

Variables	1	2	3
Number of bullfrog raised (log frogs)	·		·
Residence time (year)	·	·	
Hunting pressure	·	·	·
ΔAICc	0	0.59	0.78
AICc	−48.1	−47.5	−47.3
Wi	0.304	0.226	0.206
*R* ^*2*^	0.8691	0.8042	0.8015

•, displays that a factor is included in the model;

ΔAICc, the difference between each model and the highest ranked model;

AICc, the second-order Akaike information criterion;

Wi (Akaike weights), the probability that the predictor is a component of one of the best models;

*R*^*2*^, R-squared.

**Figure 2:**
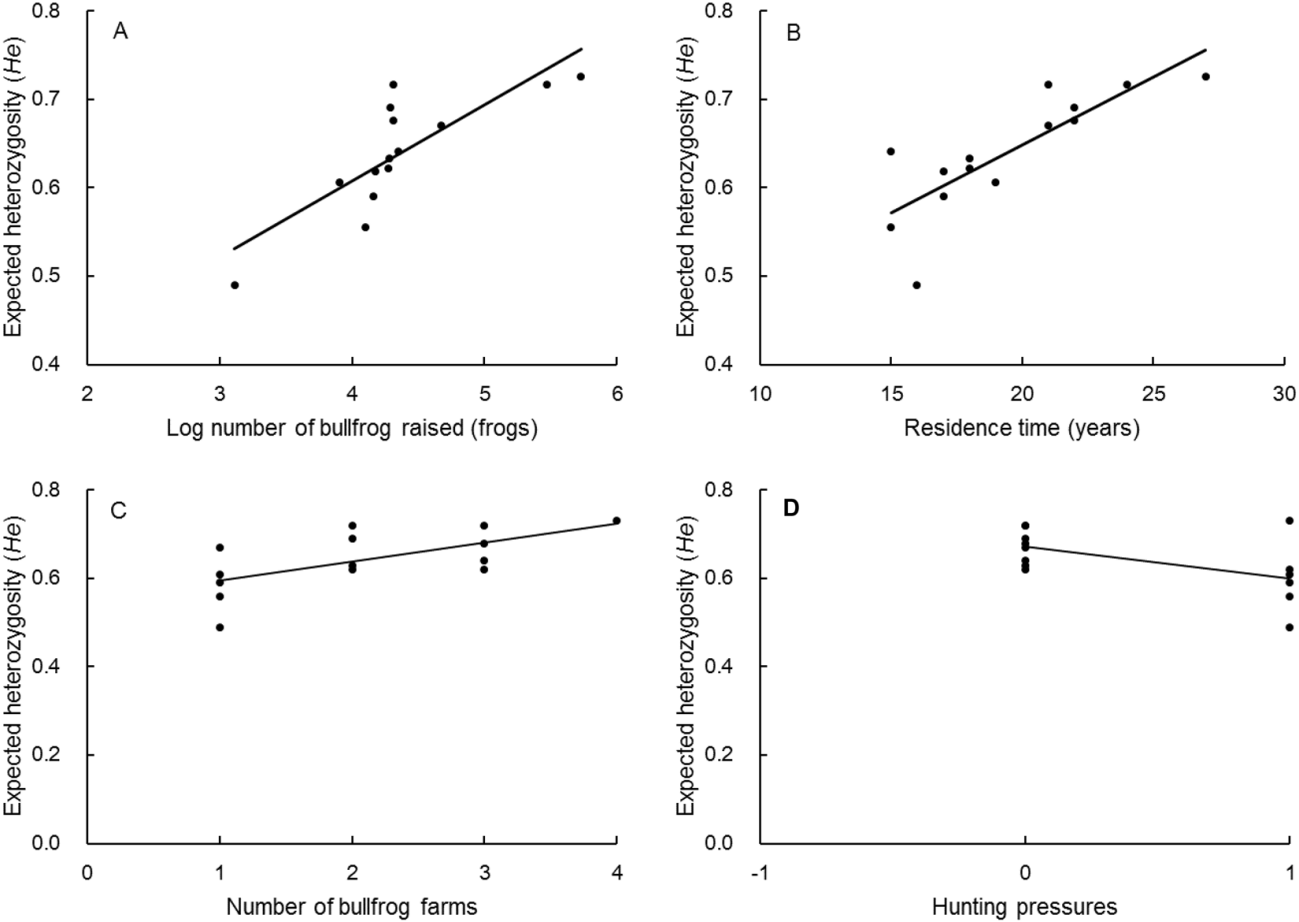
Relationships between expected heterozygosity (*He*) of bullfrog populations and predictors in islands: (**A**) number of bullfrog raised (Spearman rank correlation, *r* = 0.906, *P* < 0.001); (**B**) residence time (*r* = 0.829, P < 0.001); (**C**) number of bullfrog farms (*r* = 0.698, *P* = 0.005); and (**D**) hunting pressures (*r* = −0.574, *P* = 0.032).

The model averaging analysis also showed that the number of bullfrogs raised, the residence time and hunting pressure had high relative importance values (0.66–0.85) for expected heterozygosity in insular populations (Table 3). The model-averaged 95% confidence intervals for these variables also excluded zero. The expected heterozygosity (positive parameter) increased with increases in the number of bullfrogs raised and in the residence time for insular populations, and the expected heterozygosity (negative parameter) decreased with increases in hunting pressure for insular populations. Figure 2 shows the relationship between expected heterozygosity and these predictors. The number of bullfrog farms had lower relative importance values (0.16) for insular populations, and the model-averaged 95% confidence intervals for these variables also included zero. Similar relationships between neutral genetic variation and ecological factors were found (Tables S8–10).

**Table 3:** Summary of model averaging results based on multiple linear regression models.

Explanatory variables	β	SE	95% CI (lower, upper)	Relative importance
**Hunting pressures**	**−0.0492**	**0.0172**	**−0.0873, −0.0111**	**0.85**
**Residence time (year)**	**0.0104**	**0.0043**	**0.0015, 0.0194**	**0.71**
**Number of bullfrog raised (log frogs)**	**0.0576**	**0.0248**	**0.0053, 0.1098**	**0.66**
Number of farms	0.0147	0.012	−0.0114, 0.0409	0.16

The full model employed expected heterozygosity as the response variable and four factors as the predictors. The model-averaged 95% confidence intervals that do not overlap zero are shown in bold.

## Discussion

We detected a statistically significant pattern in which greater genetic diversity and allelic richness in feral populations of bullfrogs on the islands of the Zhoushan Archipelago than on mainland China, although some island populations (such as Jintang, Daxie and Sijiao) have genetic diversity similar to mainland populations. A feral population would have greater genetic variation if there had previously been a larger number of bullfrogs raised on farms in the invaded region or island or if bullfrogs invaded early or invaded islands. These results suggested that both the introduction history and hunting pressure likely influenced the genetic diversity in bullfrog populations.

Several studies have compared the genetic variation in introduced populations between islands and the mainland. For example, Lade *et al*.^16^ detected lower genetic variation in one insular population of *Vulpes vulpes* on Phillip Island compared to three populations on the Australian continent^16^. Similarly, higher genetic diversity was measured in eight populations of *Neovison vison* in Scotland (treated as a mainland) than one Outer Hebridean island population due to the admixture of introductions in the Scotland populations^15^. Therefore, the higher genetic diversity of bullfrog populations on the islands of the Zhoushan Archipelago than on mainland China is correlated to larger numbers of bullfrogs being raised on the islands than on the mainland and to lower hunting pressure in the islands. The propagule pressure of introductions plays an important role in determining the genetic variation of alien species^11,12,17,57,58^. The large number of bullfrogs raised may increase the number of bullfrogs escaping from the enclosure given that individual bullfrogs in the enclosures have the same chance of escaping, which would increase the input of more variation from escaped bullfrogs and therefore have positive effects on the genetic diversity of feral populations. In contrast to another study on invasive salmonids^20^, we found a positive effect of residence time on genetic variation in bullfrog populations, indicating that there might have been multiple releases at the sampled sites following the original introduction by escaped bullfrogs from farms. Bullfrog releases by both religious communities and individual persons are actively observed in China, where our sampled sites are located^33,36^. Such releases might have increased propagule pressure on the studied water bodies.

In model averaging, hunting pressure might represent the main factor that influences the survival of bullfrogs escaping from farms on the islands. Human hunting pressures on bullfrogs were lower on the Zhoushan Archipelago than in the Beilun region of the mainland likely due to smaller human populations and lower demand for frogs as food throughout much of the archipelago^10^. Lower hunting pressures would be more likely to reduce the mortality of bullfrog founders from farms on islands and could therefore could result in higher genetic diversity in insular populations than in their mainland counterparts.

Studies have found that the admixture of multiple introductions from different source populations in a native range can increase the genetic variation in the invading populations^18,19^. The higher genetic diversity in insular bullfrog populations is unlikely due to such an admixture for three reasons. First, seawater might be a significant barrier to the dispersal of bullfrogs among islands due to the species’ intolerance of seawater^30^. As a result, gene flow between island bullfrog populations might have been limited among the islands of the Zhoushan Archipelago. Second, a lack of genetic structure within all bullfrog populations indicates that multiple introductions from different source populations in the native range are unlikely to have occurred and contributed to genetic structure in each of these invasive populations. Third, this result is in line with a recent study based on the mitochondrial cytochrome *b* gene^34^, which suggests that a single introduction from a local population in the native range might have driven the limited haplotype diversity (only two haplotypes) in invading bullfrog populations in China^34,58^.

In contrast to studies that show population expansion without recent bottlenecks in invasive species^41,57,59^, we found no evidence for either recent bottlenecks or population expansion in either insular or mainland bullfrog populations (Table S6, Supporting Information), suggesting that the effective population size of bullfrog populations are stable after the establishment. Bullfrogs are characterized by their high fecundity (approximately 10,000 eggs/clutch)^60,61^, a potential that can result in high population densities and rapid population expansion. Feral bullfrog populations often meet adverse conditions in novel environments in the invaded range and cannot breed successfully in some years^28,35^, which might restrict this potential. Such demographic growth after introductions may result in the colonization of bullfrog populations with a minimal bottleneck effect or expansion effect on genetic diversity.

Both high and low levels of genetic differentiation have been observed in invasive species, including those with highly structured populations, such as *Sturnus vulgaris*^62^, *Neovison vison*^63^, *Metrioptera roeselii*^64^ and *Drosophila suzukii*^65^, and populations with a low structure, such as *Harmonia axyridis*^66^ and *Galapaganus howdenae howdenae*^57^). We detected genetic differentiation in both insular population and mainland population pairs, suggesting the existence of genetic structuring exists among bullfrog populations. However, the differentiation (*Dest*) between pairs was not related to differences in residence time (Mantel test, *r* = 0.131, *P* = 0.088), suggesting that there were no differences in genetic differentiation between old and young bullfrog populations. Three factors may be responsible for such differentiation. First, gene flow among mainland and insular populations might be very low (Table S5, Supporting Information), which would facilitate genetic differentiation among populations^67^. Similar to most amphibians^68,69^, bullfrogs are intolerant of seawater^30^, and sea barriers might isolate gene flow among insular populations. Second, large differences in the number of bullfrogs raised on farms (Table S7, Supporting Information) might lead to genetic differentiation among feral populations that originated from escaping founders. Finally, multiple releases might have an effect on the differentiation when such releases involve founders that are different from those of the first introduction^20^, which can increase or restore genetic diversity in invading populations.

The results from this study might have important implications for understanding the establishment and evolution of alien species and for the management of invasive populations on islands. Enhanced genetic variation may increase the potential for adaptive evolution, which could lead to rapid evolution by natural selection in novel environments^12,19^. Although evidence exists for successful invasions that display reductions in genetic variation^70,71^, increasing numbers of studies show that higher genetic diversity facilitates invasion success^13,14,72,73^. Higher genetic variation in insular invasive populations than in their mainland counterparts would make islands more prone to biological invasions. The available data for bullfrogs are limited but consistent with this conjecture. Bullfrogs are more likely to invade from farms located on the Zhoushan islands than from farms in the nearby the mainland^10^. Future work is required to quantify the relative contributions of ecological factors and genetic factors to the differences in the invasion success and evolution of bullfrog populations between islands and the mainland.

## Supplementary information

Supplementary Information

Supplementary Dataset

## References

[1] Simberloff D (1995). Why do introduced species appear to devastate islands more than mainland areas?. Pac Sci.

[2] Elton, C. S. The ecology of invasions by plants and animals. *Methuen, London* **18** (1958).

[3] Pysek P (2010). Disentangling the role of environmental and human pressures on biological invasions acrossEurope. P Natl Acad Sci USA.

[4] Diez JM (2009). Learning from failures: testing broad taxonomic hypotheses about plant naturalization. Ecol Lett.

[5] Clavero M, Brotons L, Pons P, Sol D (2009). Prominent role of invasive species in avian biodiversity loss. Biol Conserv.

[6] Blackburn TM, Cassey P, Lockwood JL (2008). The island biogeography of exotic bird species. Global Ecol Biogeogr.

[7] Sax DF, Gaines SD, Brown JH (2002). Species invasions exceed extinctions on islands worldwide: A comparative study of plants and birds. Am Nat.

[8] Lonsdale WM (1999). Global patterns of plant invasions and the concept of invasibility. Ecology.

[9] Case TJ (1996). Global patterns in the establishment and distribution of exotic birds. Biol Conserv.

[10] Li YM, Wu ZJ, Duncan RP (2006). Why islands are easier to invade: human influences on bullfrog invasion in the Zhoushan archipelago and neighboring mainland China. Oecologia.

[11] Rius M, Darling JA (2014). How important is intraspecific genetic admixture to the success of colonising populations?. Trends Ecol Evol.

[12] Lee CE (2002). Evolutionary genetics of invasive species. Trends Ecol Evol.

[13] Signorile AL (2014). Do founder size, genetic diversity and structure influence rates of expansion of North American grey squirrels in Europe?. Divers Distrib.

[14] Dlugosch KM, Parker IM (2008). Invading populations of an ornamental shrub show rapid life history evolution despite genetic bottlenecks. Ecol Lett.

[15] Fraser EJ, Macdonald DW, Oliver MK, Piertney S, Lambin X (2013). Using population genetic structure of an invasive mammal to target control efforts - An example of the American mink in Scotland. Biol Conserv.

[16] Lade JA, Murray ND, Marks CA, Robinson NA (1996). Microsatellite differentiation between Phillip island and mainland Australian populations of the red fox Vulpes vulpes. Mol Ecol.

[17] Peacock MM, Beard KH, O’Neill EM, Kirchoff VS, Peters MB (2009). Strong founder effects and low genetic diversity in introduced populations of Coqui frogs. Mol Ecol.

[18] Rius M, Pascual M, Turon X (2008). Phylogeography of the widespread marine invader *Microcosmus squamiger* (Ascidiacea) reveals high genetic diversity of introduced populations and non-independent colonizations. Divers Distrib.

[19] Kolbe JJ (2004). Genetic variation increases during biological invasion by a Cuban lizard. Nature.

[20] Monzon-Arguello C (2014). Contrasting patterns of genetic and phenotypic differentiation in two invasive salmonids in the southern hemisphere. Evol Appl.

[21] Wang S (2017). Pathogen richness and abundance predict patterns of adaptive MHC variation in insular amphibians. Mol Ecol.

[22] ISSG. Invasive Species Specialist Group. http://www.issg.org/database/welcome/. Accessed 6 Jun 2011 (2008).

[23] Orchard, S. Removal of the American bullfrog, *Rana (Lithobates) catesbeiana*, from a pond and a lake on Vancouver Island, British Columbia, Canada. *Island invasives: eradication and management. IUCN (Gland, Switzerland)*, 1–542 (2011).

[24] Santos-Barrera, G. *et al*. *Lithobates catesbeianus*. The IUCN Red List of Threatened Species. *Version* 2*014.3*. www.iucnredlist.org. *Downloaded on 04 December 2014* (2009).

[25] Liu X (2018). More invaders do not result in heavier impacts: The effects of non-native bullfrogs on native anurans are mitigated by high densities of non-native crayfish. J Anim Ecol.

[26] Li YM, Ke ZW, Wang SP, Smith GR, Liu X (2011). An exotic species is the favorite prey of a native enemy. Plos One.

[27] Wu ZJ, Li YM, Wang YP, Adams MJ (2005). Diet of introduced Bullfrogs (*Rana catesbeiana*): Predation on and diet overlap with native frogs on Daishan Island, China. J Herpetol.

[28] Bury, R. B. & Whelan, J. A. *Ecology and management of the bullfrog*. (US Fish and Wildlife Service Washington, DC, 1984).

[29] Garner TWJ (2006). The emerging amphibian pathogen *Batrachochytrium dendrobatidis* globally infects introduced populations of the North American bullfrog, Rana catesbeiana. Biol Letters.

[30] Wang YH, Li YM (2009). Habitat Selection by the Introduced American Bullfrog (*Lithobates catesbeianus*) on Daishan Island, China. J Herpetol.

[31] Kraus, F. *Alien reptiles and amphibians: a scientific compendium and analysis*. Vol. 4 (Springer, 2009).

[32] Ficetola GF, Thuiller W, Miaud C (2007). Prediction and validation of the potential global distribution of a problematic alien invasive species - the American bullfrog. Divers Distrib.

[33] Liu X, McGarrity ME, Bai CM, Ke ZW, Li YM (2013). Ecological knowledge reduces religious release of invasive species. Ecosphere.

[34] Bai CM, Ke ZW, Consuegra S, Liu X, Li YM (2012). The role of founder effects on the genetic structure of the invasive bullfrog (*Lithobates catesbeianaus*) in China. Biol Invasions.

[35] Li YM, Ke ZW, Wang YH, Blackburn TM (2011). Frog community responses to recent American bullfrog invasions. Curr Zool.

[36] Liu X, Li Y (2009). Aquaculture enclosures relate to the establishment of feral populations of introduced species. Plos One.

[37] Wang SP (2014). Population size and time since island isolation determine genetic diversity loss in insular frog populations. Mol Ecol.

[38] Wang S (2017). The origin of invasion of an alien frog species in Tibet, China. Curr Zool.

[39] Wang S, Liu C, Zhu W, Gao X, Li Y (2016). Tracing the origin of the black-spotted frog, *Pelophylax nigromaculatus*, in the Xinjiang Uyghur Autonomous Region. Asian Herpetol Res.

[40] Zhan, W. Isolation of microsatellite markers and 16S rRNA sequences analysis in *Rana Catesbeiana*. *Master thesis, Hunan Agricultural Universit*y (2008).

[41] Austin JD, Davila JA, Lougheed SC, Boag PT (2003). Genetic evidence for female-biased dispersal in the bullfrog, *Rana catesbeiana* (Ranidae). Mol Ecol.

[42] Van Oosterhout C, Hutchinson WF, Wills DPM, Shipley P (2004). MICRO-CHECKER: software for identifying and correcting genotyping errors in microsatellite data. Mol Ecol Notes.

[43] Raymond M, Rousset F (1995). Genepop (Version-1.2) - Population-Genetics Software for Exact Tests and Ecumenicism. J Hered.

[44] Rice WR (1989). Analyzing Tables of Statistical Tests. Evolution.

[45] Pritchard JK, Stephens M, Donnelly P (2000). Inference of population structure using multilocus genotype data. Genetics.

[46] Peakall R, Smouse PE (2012). GenAlEx 6.5: genetic analysis in Excel. Population genetic software for teaching and research-an update. Bioinformatics.

[47] Beerli P, Felsenstein J (1999). Maximum-likelihood estimation of migration rates and effective population numbers in two populations using a coalescent approach. Genetics.

[48] Jueterbock, A., Kraemer, P. & Gerlach, G. DEMEticsv0. 8.0. *University of Oldenburg, Oldenburg, Germany* (2010).

[49] Jost L (2008). GST and its relatives do not measure differentiation. Mol Ecol.

[50] Excoffier L, Laval G, Schneider S (2005). Arlequin (version 3.0): An integrated software package for population genetics data analysis. Evol Bioinform.

[51] Piry S, Luikart G, Cornuet JM (1999). BOTTLENECK: A computer program for detecting recent reductions in the effective population size using allele frequency data. J Hered.

[52] Cornuet JM, Luikart G (1996). Description and power analysis of two tests for detecting recent population bottlenecks from allele frequency data. Genetics.

[53] Bilgin R (2007). Kgtests: a simple Excel Macro program to detect signatures of population expansion using microsatellites. Mol Ecol Notes.

[54] Reich DE, Feldman MW, Goldstein DB (1999). Statistical properties of two tests that use multilocus data sets to detect population expansions. Mol Biol Evol.

[55] Burnham, K. P. & Anderson, D. R. *Model selection and multimodel inference: a practical information-theoretic approach*. (2nd edn Springer, 2002).

[56] R Development Core Team. R: A language and environment for statistical computing, R Foundation Statistical Computing, Vienna, Austria. ISBN 3-900051-07-0. Available at: http://www.R-project.org/ (2012).

[57] Mok HF, Stepien CC, Kaczmarek M, Albelo LR, Sequeira AS (2014). Genetic status and timing of a weevil introduction to Santa Cruz Island, Galpagos. J Hered.

[58] Ficetola GF, Bonin A, Miaud C (2008). Population genetics reveals origin and number of founders in a biological invasion. Mol Ecol.

[59] Zeisset I, Beebee TJC (2003). Population genetics of a successful invader: the marsh frog *Rana ridibunda* in Britain. Mol Ecol.

[60] Kaefer IL, Boelter RA, Cechin SZ (2007). Reproductive biology of the invasive bullfrog *Lithobates catesbeianus* in southern Brazil. Ann Zool Fenn.

[61] Howard RD (1978). Evolution of Mating Strategies in Bullfrogs. Rana Catesbeiana. Evolution.

[62] Rollins LA, Woolnough AP, Wilton AN, Sinclair R, Sherwin WB (2009). Invasive species can’t cover their tracks: using microsatellites to assist management of starling (*Sturnus vulgaris*) populations in Western Australia. Mol Ecol.

[63] Zalewski A (2011). High mitochondrial DNA diversity of an introduced alien carnivore: comparison of feral and ranch American mink *Neovison vison* in Poland. Divers Distrib.

[64] Kanuch P, Berggren A, Cassel-Lundhagen A (2013). Colonization history of *Metrioptera roeselii* in northern Europe indicates human-mediated dispersal. J Biogeogr.

[65] Adrion JR (2014). Drosophila suzukii: The Genetic Footprint of a Recent, Worldwide Invasion. Mol Biol Evol.

[66] Lombaert E (2014). Rapid increase in dispersal during range expansion in the invasive ladybird *Harmonia axyridis*. J Evolution Biol.

[67] Huang, B. H., Huang, C. W., Huang, C. L. & Liao, P. C. Continuation of the genetic divergence of ecological speciation by spatial environmental heterogeneity in island endemic plants. *Sci Rep-Uk* **7**, 10.1038/s41598-017-05900-1 (2017). 10.1038/s41598-017-05900-1 PMC5511155 28710389

[68] Duellman, W. E. & Trueb, L. *Biology of amphibians*. (Johns Hopkins University Press, 1994).

[69] Balinsky JB (1981). Adaptation of Nitrogen-Metabolism to Hyperosmotic Environment in Amphibia. J Exp Zool.

[70] Tsutsui ND, Suarez AV, Holway DA, Case TJ (2000). Reduced genetic variation and the success of an invasive species. P Natl Acad Sci USA.

[71] Queller DC (2000). Evolutionary ecology: Pax Argentinica. Nature.

[72] Wang XY (2012). Genotypic diversity enhances invasive ability of *Spartina alterniflora*. Mol Ecol.

[73] Crawford KM, Whitney KD (2010). Population genetic diversity influences colonization success. Mol Ecol.

