# Supplementary material for: Propagule pressure and hunting pressure jointly determine genetic evolution in insular populations of a global frog invader
Source: Sci Rep. 2019 Jan 24;9:448. doi: 10.1038/s41598-018-37007-6 (PMC6345768; doi:10.1038/s41598-018-37007-6)

Supporting information

Propagule pressure and hunting pressure jointly determine genetic evolution in insular populations of a global frog invader

Supen Wang, Conghui Liu, Jun Wu, Chunxia Xu, Jiaqi Zhang, Changming Bai, Xu Gao, Xuan Liu, Xianping Li, Wei Zhu, and Yiming Li

Table S1 Questionnaire survey method and forms used to assess the bullfrog residence time.

Table S2 Information of all the primers used in the study.

Table S3 Population sampling localities, GPS coordinates, and sample sizes (n).

Table S4 Pairwise *Dest* values for populations of *Lithobates catesbeianaus*.

Table S5 Asymmetric migration rates between *Lithobates catesbeianaus* populations inferred with MIGRATE. The populations in bold and italics are the source and sink populations, respectively. The data are presented as mean values (±95% CIs).

Table S6 Detection of the population expansion of *Lithobates catesbeianaus*.

Table S7 Predictors for the genetic diversity of bullfrog populations

Table S8 The best models (i.e. ΔAIC $\leq$ 2) containing factors influencing the genetic variation of *L. catesbeianaus* in the Zhoushan Archipelago.

Table S9 Summary of model averaging results based on multiple linear regression models.

Table S10 Coefficients and probabilities of the Spearman correlation analysis among predictors.

Figure S1 Genetic clusters (K) obtained from the STRUCTURE analysis of three invasive regions of the mainland and 14 islands.

Figure S2 Coefficients and probabilities (in parentheses) of the Spearman correlation analysis among predictors.

Table S1

Questionnaire survey method on bullfrog residence time and Questionnaire Form

Methods

A questionnaire was sent to residents of houses on or close to the banks of water bodies, or to farmers that live near a water body and had raised bullfrogs on their farms before, or to patrolmen who are responsible for the safety of a water body. The residents were most likely first to sight bullfrog individuals or hear bullfrog calls when bullfrogs invaded water bodies in close proximity; the farmers were likely to notice if their bullfrogs had escaped from their farms, and to where; the patrolmen usually patrolled the banks of a water body once every one to two weeks, and so were likely to notice any unusual events at the water bodies, including sight or sound of bullfrogs. People canvassed were usually at least 45 years old (this means that they were already adults in the period when local farms started raising bullfrogs), and long-term workers or residents in the village where the water bodies investigated were located. Most of the people knew farmers when the farmers in their village were raising bullfrogs.

People were first asked if they recognized bullfrogs by describing the morphology and let them see pictures of bullfrogs or if they recognized calls of bullfrogs by play backing the calls. Those who did not recognize bullfrogs or their calls were excluded from the further survey. People were then asked if they had sighted bullfrog individuals or heard bullfrog calls, and for how many years, in a sampled water body that they lived close to or that they patrolled. They were also asked to describe distinct events linked to the time when they first saw bullfrogs. This would help to clarify if the accuracy of their answers could be confirmed by independent events. Answers that could not be confirmed by events were excluded from the analysis.

Questionnaire Form

1. Basic information from interviewers

A. Surname:

:

B. Age (about, can be estimated by investigators):

C. Periods that you live around the water body investigated:

D: Job:

E. Working experience in other villages or cities

2. Questions for interviewers

A: Do you know who ever raised bullfrog before in your villages or other villages?

B. Do you recognize American bullfrogs or bullfrogs?

C. What the bullfrogs look like or what bullfrog calls sound like?

D. When did you first see bullfrogs or hear bullfrog calls from the water body investigated?

E. Why are you sure the year that you saw bullfrogs or heard bullfrog calls from the water body? Were there some significant events occurring that year (significantly political events, large natural disasters, the year that your child went to some schools, birth dates or death date of your family members or your neighbor hoods, periods that someone lost a job that was related to you, and so on)?

Table S2

Characteristics of the nine microsatellite loci used in the study

Locus	Primer sequence (5’-3’′)	Repeat motif	Length (bp)	*Ta* (℃)	Reference
Rcat3–2b	F: CACCCAACTAATTAGGATGGG	$(AC)_2AT(AC)_{12}$	144	62	Austin *et al*. 2003
	R: GGAATGGCATTCAGAGAGGG				
Rcat J8	F: CCATAGGAATCAAAACAACCCTC	$(GT)_{18}$	100	58	Austin *et al*. 2003
	R: GGGATATGTGATGGACCCAAG				
Rcat J44b	F: AGGTTAATGAAGCTCGGCAG	$(GT)_{22}$	93	60	Austin *et al*. 2003
	R: GGAGGCATCATATCAGAGAG				
Rcat J54	F: TCATTACCACTGCCTTCTGC	$(CA)_{18}$	183	60	Austin *et al*. 2003
	R: TGCTGCTGTCCTATTGCTAG				
Rcat J41	F: CAGCAATTGGGACTCCATAAG	$(CA)_{20}$	243	62	Austin *et al*. 2003
	R: AGGTCGACTCTAGCAGAAGC				
Rcat J21	F: CCCATCTTATCCTGTGTACT	$(GT)_{25}$	177	58	Austin *et al*. 2003
	R: CAAGCCTCCATCTCACCTTACC				
Rcat J11	F: CTCTTCCATTCACAACTGCAC	$(CA)_{16}C(CA)_3$	124	60	Austin *et al*. 2003
	R: AGGCGGAGCATATATTGTGG				
BF1	F: TTTAAGTACCGTAGTTTG	$(CT)_{14}$	119	56.5	Zhan 2008
	R:TGCTAGAAAATTAGAAACT				
BFDll	F: CGAACATGAAAAAAGTGT	$(GT)_{29}$	101	45	Zhan 2008
	R: ATTCATTCTTCTCAGACT				

Table S3

Population sampling localities, GPS coordinates, sample sizes (n)

Locations	Sampling sites	GPS		sample sizes (n)
**Mainland**				
Wuhu	1	31 °3.365'	118 °18.748'	30
Beilun	1	29 °51.356'	121 °59.833'	15
	1	29 °49.640'	121 °54.905'	15
Yangzhou	1	32 °21.916'	119 °9.980'	7
	2	32 °21.687'	119 °9.465'	8
	3	32 °21.269'	119 °9.703'	7
	4	32 °21.557'	119 °8.826'	8
**Islands**				
Cezi	1	30 °5.687'	121 °55.454'	11
	2	30 °5.701'	121 °55.966'	9
	3	30 °5.315'	121 °56.550'	10
Daishan	1	30 °19.019'	122 °10.966'	7
	2	30 °18.654'	122 °12.022'	7
	3	30 °18.093'	122 °11.008'	8
	4	30 °17.514'	122 °10.840'	8
Dapengshan	1	30 °4.322'	121 °50.227'	30
Daxie	1	29 °54.168'	121 °56.929'	15
	2	29 °54.747'	121 °56.994'	15
Dengbu	1	29 °52.685'	122 °18.081'	18
	2	29 °51.781'	122 °18.198'	12
Fodu	1	29 °44.830'	122 °1.407'	30
Jintang	1	30 °3.575'	121 °51.487'	17
	2	30 °3.096'	121 °52.298'	13
Liuheng	1	29 °45.023'	122 °6.548'	15
	2	29 °43.749'	122 °7.635'	7
	3	29 °42.644'	122 °8.695'	8
Sijiao	1	30 °43.190'	122 °27.601'	15
	2	30 °42.804'	122 °27.526'	15
Taohua	1	29 °49.676'	122 °15.654'	10
	2	29 °49.481'	122 °16.807'	10
	3	29 °49.034'	122 °16.941'	10
Xiazhi	1	29 °46.256'	122 °13.637'	14
	2	29 °45.898'	122 °14.422'	16
Xiushan	1	30 °10.842'	122 °9.708'	10
	2	30 °11.114'	122 °10.566'	10
	3	30 °10.367'	122 °10.010'	10
Zhoushan	1	30 °1.017'	122 °17.017'	11
	2	30 °3.990'	122 °7.779'	9
	3	30 °5.783'	122 °2.958'	10
Zhujiajian	1	29 °55.770'	122 °21.340'	30

Table S4

Pairwise *Dest* values for populations of *Lithobates catesbeianaus* (below diagonals) and and corresponding *P*-values (above diagonals).

	Wuhu	Beilun	Yangzhou	Dapengshan	Liuheng	Taohua	Dengbu	Zhujiajian	Zhoushan	Xiushan	Daishan	Xiazhi	Fodu	Cezi	Jintang	Daxie	Sijiao
Beilun	0.634		**	**	**	**	**	**	**	**	**	**	**	**	**	**	**
Yangzhou	0.48	0.55		**	**	**	**	**	**	**	**	**	**	**	**	**	**
Dapengshan	0.454	0.624	0.51		**	**	**	**	**	**	**	**	**	**	**	**	**
Liuheng	0.425	0.489	0.407	0.286		**	**	**	**	**	**	**	**	**	**	**	**
Taohua	0.619	0.49	0.603	0.587	0.338		**	**	**	**	**	**	**	**	**	**	**
Dengbu	0.51	0.519	0.47	0.5	0.325	0.339		**	**	**	**	**	**	**	**	**	**
Zhujiajian	0.425	0.511	0.464	0.313	0.231	0.467	0.364		**	**	**	**	**	**	**	**	**
Zhoushan	0.391	0.432	0.437	0.335	0.179	0.336	0.343	0.304		**	**	**	**	**	**	**	**
Xiushan	0.442	0.285	0.432	0.397	0.26	0.381	0.324	0.243	0.266		**	**	**	**	**	**	**
Daishan	0.546	0.448	0.558	0.413	0.344	0.338	0.375	0.291	0.294	0.196		**	**	**	**	**	**
Xiazhi	0.7	0.432	0.643	0.404	0.391	0.502	0.493	0.448	0.41	0.337	0.175		**	**	**	**	**
Fodu	0.476	0.511	0.484	0.289	0.405	0.519	0.466	0.42	0.418	0.408	0.443	0.476		**	**	**	**
Cezi	0.474	0.523	0.458	0.525	0.385	0.401	0.47	0.426	0.384	0.432	0.469	0.631	0.263		**	**	**
Jintang	0.551	0.56	0.624	0.645	0.457	0.273	0.431	0.508	0.37	0.492	0.473	0.628	0.598	0.254		**	**
Daxie	0.532	0.535	0.611	0.652	0.521	0.282	0.422	0.496	0.416	0.486	0.428	0.627	0.546	0.256	0.037		**
Sijiao	0.632	0.556	0.559	0.727	0.48	0.244	0.433	0.533	0.423	0.494	0.465	0.659	0.568	0.207	0.102	0.107	

P value after the Benjamini–Hochberg correction for multiple tests (***P*<0.01).

Table S5

Asymmetric migration rates between *Lithobates catesbeianaus* populations inferred in MIGRATE. Populations in bold and italics are the source and sink populations respectively. Mean value (±95% CIs). Migration rates (*M*=m/μ, where m is the immigration rate per generation) have been estimated between populations using a coalescent based Monte Carlo Markov Chain method. We used a Brownian approximation model, and mutation was considered to be constant for all loci. We implemented *Fst* estimates and a UPGMA tree as starting parameters for the estimation of M and performed five independent runs using one long chain with a run of 10^7 recorded parameter genealogies after discarding the first 10^5 genealogies as burn-in for each locus.

	Wuhu	*Beilun*	*Yangzhou*	*Dapengshan*	*Liuheng*	*Taohua*	*Dengbu*	*Zhujiajian*	*Zhoushan*	*Xiushan*	*Daishan*	*Xiazhi*	*Fodu*	*Cezi*	*Jintang*	*Daxie*	*Sijiao*
**Beilun**	3(0-17)		3(0-17	3(0-17)	3(0-17)	3(0-17)	3(0-18)	7(0-24)	3(0-17)	5(0-21)	3(0-17)	3(0-17)	4(0-18)	2(0-16)	3(0-17)	3(0-17)	2(0-16)
**Yangzhou**	4(0-18)	3(0-16)		3(0-17)	6(0-21)	2(0-16)	5(0-20)	3(0-17)	3(0-17)	4(0-18)	3(0-17)	3(0-17)	3(0-17)	3(0-17)	3(0-18)	2(0-16)	5(0-21)
**Dapengshan**	3(0-17)	4(0-18)	3(0-17)		4(0-19)	3(0-17)	2(0-16)	5(0-21)	6(0-22)	3(0-17)	3(0-17)	2(0-16)	4(0-19)	3(0-17)	4(0-19)	3(0-17)	4(0-18)
**Liuheng**	4(0-19)	3(0-17)	3(0-17)	3(0-18)		4(0-18)	3(0-17)	3(0-17)	4(0-18)	4(0-19)	3(0-17)	3(0-17)	3(0-19)	2(0-15)	3(0-17)	3(0-17)	3(0-17)
**Taohua**	2(0-16)	3(0-17)	4(0-19)	4(0-19)	3(0-17)		4(0-18)	3(0-17)	4(0-19)	3(0-17)	4(0-19)	3(0-17)	3(0-17)	3(0-17)	4(0-19)	3(0-17)	3(0-17)
**Dengbu**	3(0-17)	4(0-18)	5(0-20)	7(0-23)	3(0-17)	3(0-18)		3(0-17)	4(0-18)	3(0-17)	4(0-19)	6(0-22)	2(0-16)	2(0-16)	3(0-17)	3(0-17)	3(0-17)
**Zhujiajian**	3(0-18)	3(0-17)	4(0-19)	4(0-20)	3(0-17)	3(0-17)	4(0-19)		7(0-24)	4(0-18)	4(0-18)	4(0-19)	3(0-17)	4(0-19)	4(0-18)	2(0-16)	2(0-15)
**Zhoushan**	3(0-17)	3(0-17)	3(0-17)	3(0-17)	6(0-22)	2(0-16)	3(0-18)	3(0-18)		4(0-19)	3(0-17)	4(0-18)	4(0-19)	3(0-17)	5(0-21)	3(0-17)	3(0-17)
**Xiushan**	2(0-16)	3(0-17)	3(0-17)	4(0-18)	7(0-25)	3(0-17)	2(0-16)	3(0-18)	3(0-17)		3(0-18)	2(0-16)	2(0-16)	3(0-17)	4(0-18)	3(0-18)	3(0-17)
**Daishan**	3(0-17)	3(0-16)	3(0-17)	5(0-20)	3(0-17)	4(0-18)	4(0-19)	3(0-17)	5(0-21)	4(0-19)		3(0-18)	5(0-21)	4(0-19)	4(0-19)	4(0-19)	3(0-17)
**Xiazhi**	3(0-17)	5(0-21)	3(0-17)	3(0-17)	3(0-17)	4(0-20)	4(0-19)	3(0-17)	4(0-18)	3(0-17)	5(0-20)		4(0-19)	3(0-18)	4(0-19)	3(0-18)	3(0-17)
**Fodu**	3(0-17)	2(0-16)	4(0-18)	4(0-19)	3(0-17)	3(0-17)	4(0-19)	4(0-19)	5(0-21)	4(0-19)	3(0-17)	4(0-19)		6(0-22)	3(0-17)	4(0-18)	3(0-17)
**Cezi**	3(0-17)	3(0-17)	3(0-17)	3(0-17)	5(0-21)	4(0-20)	3(0-17)	3(0-17)	3(0-17)	5(0-21)	4(0-19)	3(0-17)	3(0-17)		3(0-17)	3(0-18)	4(0-19)
**Jintang**	5(0-21)	2(0-15)	2(0-16)	5(0-20)	2(0-16)	2(0-16)	5(0-21)	3(0-17)	3(0-17)	3(0-17)	3(0-18)	2(0-16)	2(0-16)	6(0-21)		3(0-17)	3(0-17)
**Daxie**	3(0-17)	3(0-17)	3(0-17)	4(0-19)	3(0-17)	4(0-19)	2(0-16)	3(0-17)	5(0-21)	4(0-19)	3(0-17)	3(0-17)	3(0-17)	4(0-18)	3(0-21)		3(0-17)
**Sijiao**	3(0-17)	4(-019)	3(0-17)	3(0-17)	3(0-17)	2(0-16)	2(0-16)	3(0-17)	5(0-21)	3(0-17)	3(0-17)	2(0-16)	3(0-18)	4(0-19)	6(0-22)	3(0-17)	

Table S6

Tests of population expansion in feral bullfrog populations using the *k*- and *g*-tests for nine microsatellite loci

Sites	*k*-Test (number of negatives)	*g*-Test
**Mainland**		
Wuhu	5/9 NS	1.51
Beilun	5/9 NS	1.56
Yangzhou	5/9 NS	0.65
**Islands**		
Dapengshan	2/9 NS	0.61
Liuheng	2/9 NS	0.9
Taohua	3/9 NS	0.77
Dengbu	1/9 NS	0.95
Zhujiajian	4/9 NS	1.45
Zhoushan	4/9 NS	0.82
Xiushan	3/9 NS	1.22
Daishan	3/9 NS	1.23
Xiazhi	2/9 NS	0.83
Fodu	2/9 NS	0.63
Cezi	2/9 NS	0.81
Jintang	4/9 NS	1.1
Daxie	2/9 NS	0.75
Sijiao	4/9 NS	2.04

Table S7

Predictors for genetic diversity of bullfrog populations: Area= island area, NBR= Number of bullfrog raised, RT= Residence time, NF = Number of farms, HP = hunting pressures

Location	NBR (frogs)	RT (years)	NF	HP	Area (km^2)
**Mainland**					
Beilun	14400	23	2	1	
Wuhu	7900	19	1	1	
Yangzhou	10000	22	1	1	
**Islands**					
Cezi	22240	15	3	0	14.2
Daishan	20490	22	3	0	105
Dapengshan	14930	17	2	1	3.9
Daxie	12570	15	1	1	30.8
Dengbu	47090	21	1	0	15.9
Fodu	18750	18	3	0	7.1
Jintang	14590	17	1	1	76.6
Liuheng	20490	21	3	0	92.9
Sijiao	1300	16	1	1	21.3
Taohua	19220	18	2	0	40.1
Xiazhi	8000	19	1	1	16.4
Xiushan	297850	24	2	0	22.9
Zhoushan	533330	27	4	1	501.8
Zhujiajian	19310	22	2	0	60.9

Table S8-1.

The best models (i.e. ΔAIC ≤ 2) containing factors influencing the genetic variation (*Ho*) of *L. catesbeianaus* in the Zhoushan Archipelago.

Variables	1	2
Number of bullfrog raised (log frogs)		
Residence time (year)	•	•
Number of farms		
Hunting pressure		•
ΔAICc	0	1.87
AICc	-35.1	-33.2
Wi	0.41	0.16
R^2	0.5	0.58

•, displays that a factor is included in the model;
ΔAICc, the difference between each model and the highest ranked model;
AICc, the second-order Akaike information criterion;
Wi (Akaike weights), the probability that the predictor is a component of one of the best models;
R^2, R-squared.

Table S9-1.

A summary of model averaging based on multiple linear regression models. The full model employed *Ho* as the response variable and four factors as predictors. Model-averaged 95% confidence intervals that do not overlap zero are shown in bold.

Explanatory variables	β	SE	95% CI (lower, upper)	Relative importance
Hunting pressures	-0.0413	0.0316	-0.1108, 0.0282	0.25
**Residence time (year)**	**0.0141**	**0.005**	**0.0033, 0.0249**	**0.77**
Number of bullfrog raised (log frogs)	0.0547	0.0401	-0.0302, 0.1396	0.27
Number of farms	0.0185	0.0205	-0.025, 0.0629	0.16

Table S8-2.

The best models (i.e. ΔAIC ≤ 2) containing factors influencing the genetic variation (*Na*) of *L. catesbeianaus* in the Zhoushan Archipelago.

Variables	
Number of bullfrog raised (log frogs)	
Residence time (year)	•
Number of farms	
Hunting pressure	•
ΔAICc	0
AICc	23.5
Wi	0.51
R^2	0.81

•, displays that a factor is included in the model;
ΔAICc, the difference between each model and the highest ranked model;
AICc, the second-order Akaike information criterion;
Wi (Akaike weights), the probability that the predictor is a component of one of the best models;
R^2, R-squared.

Table S9-2.

A summary of model averaging based on multiple linear regression models. The full model employed *Na* as the response variable and four factors as predictors. Model-averaged 95% confidence intervals that do not overlap zero are shown in bold.

Explanatory variables	β	SE	95% CI (lower, upper)	Relative importance
Hunting pressures	**-0.54**	**0.23**	**-1.0469, -0.0332**	**0.66**
**Residence time (year)**	**0.1845**	**0.0393**	**0.0986, 0.2703**	**0.99**
Number of bullfrog raised (log frogs)	0.2024	0.1486	-0.6099, 1.049	0.1
Number of farms	0.2195	0.3894	-0.1231, 0.5278	0.23

Table S8-3.

The best models (i.e. ΔAIC ≤ 2) containing factors influencing the effective population size of *L. catesbeianaus* in the Zhoushan Archipelago.

Variables	1	2
Number of bullfrog raised (log frogs)		
Residence time (year)	•	•
Number of farms		•
Hunting pressure		
ΔAICc	0	1.64
AICc	-66.5	-64.9
Wi	0.55	0.24
R^2	0.78	0.81

•, displays that a factor is included in the model;
ΔAICc, the difference between each model and the highest ranked model;
AICc, the second-order Akaike information criterion;
Wi (Akaike weights), the probability that the predictor is a component of one of the best models;
R^2, R-squared.

Table S9-3.

A summary of model averaging based on multiple linear regression models. The full model employed effective population size as the response variable and four factors as predictors. Model-averaged 95% confidence intervals that do not overlap zero are shown in bold.

Explanatory variables	β	SE	95% CI (lower, upper)	Relative importance
Hunting pressures	-0.0043	0.0105	-0.0275, 0.0189	0.11
**Residence time (year)**	**0.0087**	**0.0016**	**0.0052, 0.0123**	**1**
Number of bullfrog raised (log frogs)	0.0007	0.0143	-0.0304, 0.0317	0.1
Number of farms	0.008	0.0056	-0.0044, 0.0203	0.28

Table S10.

The relationships between the genetic variation of bullfrog populations and predictors in islands. Observed heterozygosity: *Ho*; mean number of alleles: *Na*; effective population size: θ.

		Na	*Ho*	θ
Hunting pressure				
	r	-0.556	-0.269	-0.43
	P	0.039	0.353	0.125
Number of bullfrog raised (log frogs)				
	r	0.767	0.566	0.654
	P	0.001	0.035	0.011
Residence time (year)				
	r	0.885	0.843	0.833
	P	<0.001	<0.001	<0.001
Number of farms				
	r	0.536	0.38	0.751
	P	0.048	0.18	0.002

Figure S1

The approach was run using an admixture model estimating the most likely number of genetic clusters (K) populations that have different allele frequencies at a number of independent loci. The burn in was set to 500,000 iterations followed by 1,000,000 iterations of MCMC. All runs were repeated ten times for each number of possible genetic clusters (K) ranging from 1 to n+1(n is number of sampling sites in different regions of mainland or islands). The optimal number of clusters (the best K) was determined using method of [1] and was implemented in STRUCTURE HARVESTER [2]. The ΔK method was not applicable in this dataset because mean lnP(X|K) decreased and its variance increased with increasing values of K [1].

a:Beilun; b:Wuhu; c:Yangzhou; d:Cezi; e:Daishan; f:Daxie; g:Dengbu; h:Jingtang; i:Liuheng; j:Sijiao; k:Taohua; l:Xiashi; m:Xiushan; n:Zhoushan

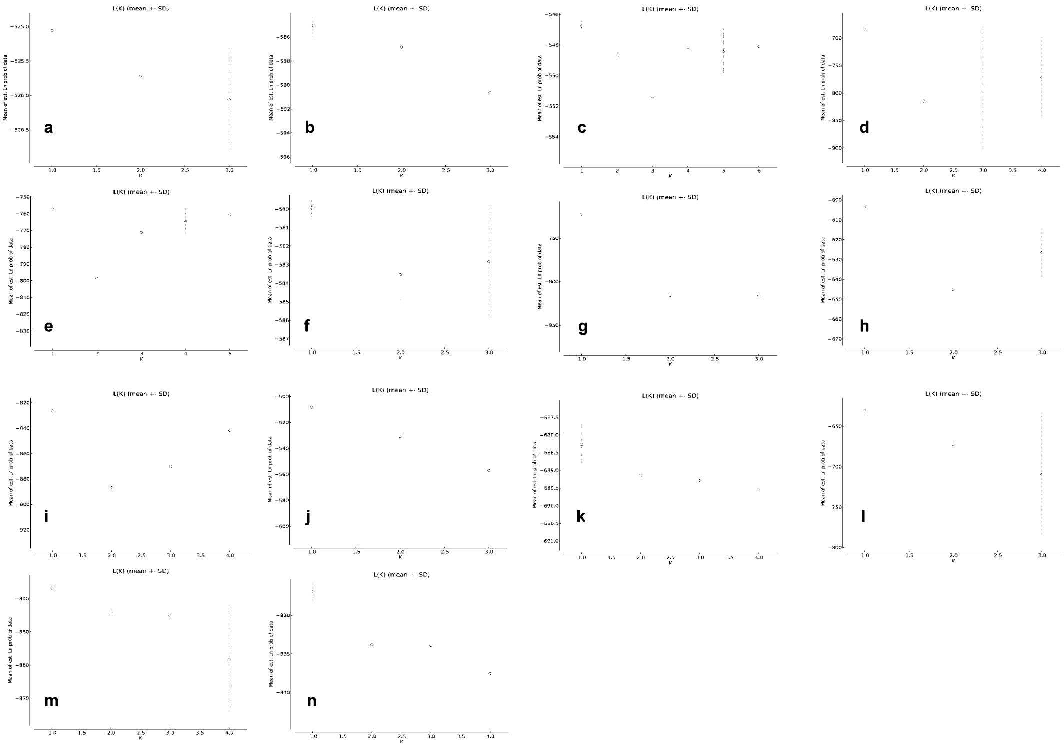

1 Evanno, G., Regnaut, S. & Goudet, J. Detecting the number of clusters of individuals using the software STRUCTURE: a simulation study. *Mol Ecol* **14**, 2611-2620, doi:DOI 10.1111/j.1365-294X.2005.02553.x (2005).

2 Earl, D. A. & Vonholdt, B. M. STRUCTURE HARVESTER: a website and program for visualizing STRUCTURE output and implementing the Evanno method. *Conserv Genet Resour* **4**, 359-361, doi:DOI 10.1007/s12686-011-9548-7 (2012).

Figure S2

The relationships among predictors: A, number of bullfrogs raised and number of farms for insular populations (Spearman correlation, r = 0.627, P = 0.016); B, number of bullfrogs raised and residence time for insular populations (r = 0.655, P = 0.011); C, number of bullfrogs raised and hunting pressures for insular populations (r = -0.574, P = 0.032).

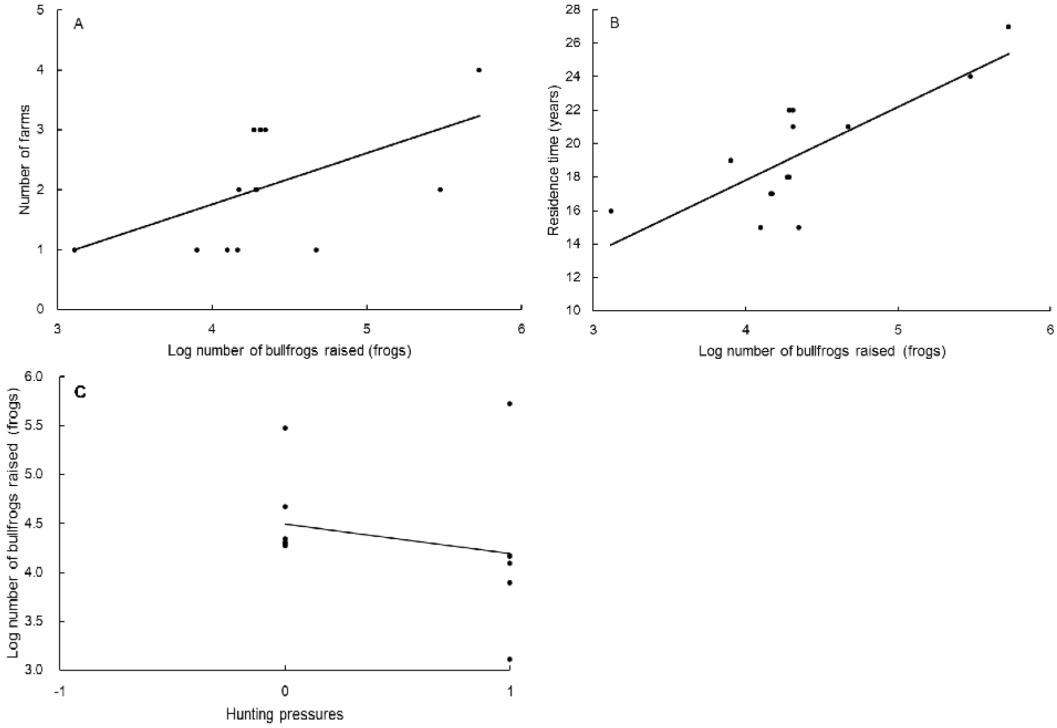

Supplement: Supplementary file 1 — Supplementary Information [file 41598_2018_37007_MOESM1_ESM.pdf]
